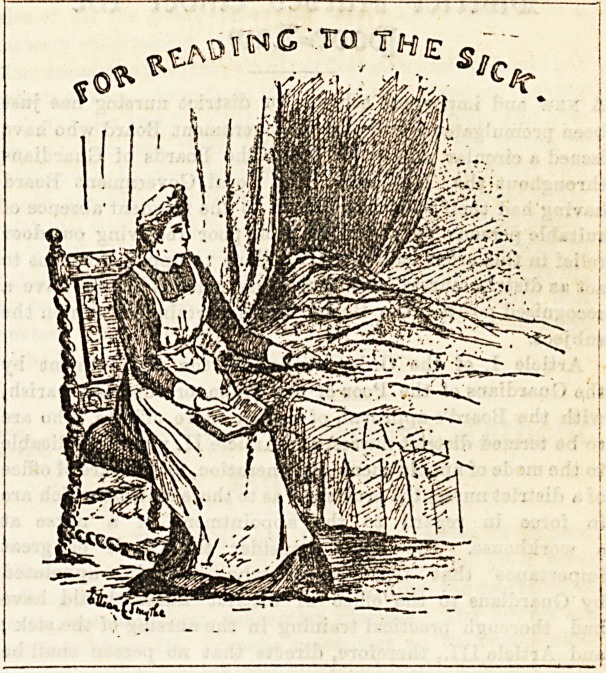# The Hospital Nursing Supplement

**Published:** 1892-02-20

**Authors:** 


### "The Hospital" Nursing Mirror.

Being the Extra Nursing Supplement of "The Hospital" Newspaper.

Contributions for this Supplement should be addressed to the Editor. The Hospital, 140, Strand, London, W.C., and should have the word "Nursing" plainly written in left-hand top corner of the envelope.

### En Passant.

**ANOTHER HOME.**—Broadstairs is shortly to boast a nursing home, over which Miss Courtenay Clarke, now Matron at the Yeatman Hospital, Sherborne, will rule. Miss Courtenay Clarke only means the Broadstairs arrangements to be temporary, and will shortly move nearer London. Nurses for private cases will be sent out in March. A good many invalids go to Broadstairs, and the scheme ought to succeed.

**SHORT ITEMS.**—Miss Wedgewood seems to be a great favourite with the Royal Free Nurses, even with those old nurses whom one expects to resent a new Matron.—Nurse Lee, late of Chertsey, went to the Hull Sanatorium on January 25th, and had to leave on February 6th.—The new home for the district nurses of Leeds is to be called the Clarence Home, in memory of the late Duke of Clarence.—Lady Zetland is ever forward in good works; she has taken under her charge some of the wretched victims of the Curragh Orphanage, and she has been inspecting the Dublin workhouses.—Dr. Duke, of Eastbourne, lately turned a nurse, whom he suspected of meddling with the patient's brandy, out of the house; the nurse took legal proceedings, but has lost her case.—St. John's District nurses at Worcester paid 12,000 visits last year; the Dean presided at the late annual meeting.—The widow mentioned in "Our Indian Letter" for December 19th has found work, and thanks the various Matrons who sent her particulars of their institutions.—*The Lady* is just now addressing some sensible articles to those who wish to become nurses.—Worthing records a satisfactory first year of district nursing, and appeals for funds to make the work permanent.—The Mirfield Society for Nursing the Sick Poor held a successful meeting on February 2nd.—Berwick means to have a district nurse, and has deputed a committee to carry out their wish.—A great gathering of nurses is expected at Westminster Hospital on Wednesday evening at eight o'clock, when the Master of St. Katharine's will discourse on the Queen's nurses.

**WORKHOUSE INFIRMARY NURSES.**—Before us lies the twelfth report of the Workhouse Infirmary Nursing Association, telling of a tremendous amount of work done with a small income of £730. The Association has 3 Superintendants and 100 nurses at work, and has 31 probationers in training; and all these are working to grant a little ease to those worsted in the battle of life—to the dying paupers to whom the very bitterest dregs of poverty and sickness have come. Still there are many union infirmaries where untrained women or even imbeciles are employed as nurses, but as the Association gains in power, these cases decrease. The report says: "We have received 126 applications, and have been able to supply 81 nurses; of these 34 were trained by the Association during the year. We find that probationers trained by ourselves are more and more in demand. This is shown by the fact that in many cases Boards of Guardians have preferred to wait some months until our probationers have completed their training. That we are able at all adequately to meet this increasing demand is largely due to the generosity of Lady Wantage and Miss Twining, who have contributed this year special donations of £100 each." The northern branch is now in working order, the Hon. Secretary being Mrs. Graham Steele, Upper Chorlton Road, Manchester. An earnest appeal is made to all enlightened Boards of Guardians for more extensive support.

**WOMEN AS ARMY NURSES.**—Under this title there was a long and interesting article in last week's *Queen* giving the particulars which bind Her Majesty's Nursing Sisters. The writer states that Lady Superintendents are paid from £150 to £200 a-year, and Sisters from £30 to £50. They have to be between 25 and 35 years of age when they enter the service, and they have to retire on a pension at the age of 60. With regard to the Female Indian Nursing Service, the writer is somewhat at fault, as instead of 25 there are now 50 Sisters at work in India, this branch having proved the most popular in the service. The Indian Sisters get Rs.175 a-month, £15 outfit money, and their passage money. They have to sign for five years.

**AMERICAN DISTRICT NURSES.**—In this month's number of the *Charities Review*, which is the organ of the New York Charity Organization Society, is an interesting paper, by Miss Isabel Hampton, on "District Nursing." Miss Hampton is Superintendent of Nurses at the John Hopkins Hospital, Baltimore, and last year she visited England, and amongst other inspections spent a morning with the North London Nursing Association. There is many a lesson which John Bull can yet teach Jonathan, many a lesson perhaps which we may have to learn from Jonathan, but in district nursing we are far in advance of America. So Miss Hampton having seen how we did things here, went back and gave a very clever account of what she had seen, and urged for as wide support there as here for nurses for the poor. The Boston Association supplies nurses to each dispensary district, of which there are six in that town; Chicago has a flourishing Visiting Nurses' Association, also employing six nurses, who work under relief committees; Philadelphia has something of the same kind, and in New York are a few scattered nurses working in connection with different churches. But the whole is poor compared with the English system now consolidated by the Jubilee gift. In the same paper is an account of the lately started "Hull House," which is the first American experiment on the same lines as our Toynbee Hall.

**CANTERBURY INSTITUTE.**—Last year was a somewhat troubled one at this Institute, but happily all difficulties are now over, and a good report is issued. The finances are not very flourishing, but as this is caused by the just proceeding of raising the wages of the nurses, and procuring a better and more highly trained class, we are sure the public will also raise their subscriptions. The new district nurse gives great satisfaction, but there is room for a second, as the cases are increasing rapidly. Says the report: "The House Committee have had to encounter certain difficulties in connection with the resignation of the late Lady Superintendent, but they are happy to find that her successor, by her tact and energy in taking up a new and somewhat difficult post at a critical moment, has more than justified the high testimonials which led to her appointment, and they confidently appeal to the subscribers of the Institute to give her all the support in their power. Enquiries which have been made show that to make an institute such as ours a real success and self-supporting, it is absolutely essential to have a staff of at least from 20 to 25 nurses, and those fully qualified by proper training, to rank as first-class nurses. Our number has heretofore never reached 14, exclusive of our district nurse, and most of our nurses have not had sufficient training or have not been fully qualified. We have, therefore, increased the number of our probationers. We have given them salaries, not hitherto done, and are providing them with a longer period of training. We have also increased the salaries of our nurses."

### Lectures on Surgical Ward Work and Nursing.

By Alexander Miles, M.D. (Edin.), F.R.C.S.E.

### Lecture XLV.—INSTRUMENTS.

Various forms and sizes of bougie are used for the œsophagus as for other canals. Some of these are of uniform calibre and are made of soft gum-elastic. Others consist of ivory or silver olive-shaped balls, fixed on to a whalebone rod, and graduated to a scale. They are used for purposes of diagnosis and treatment. A combined probang and coin-catcher illustrated in Fig. 2, and an umbrella-probang in Fig. 3. This latter consists of a bunch of bristles which is passed into the œsophagus, and then by a mechanical arrangement opened out, so that on being withdrawn it brushes any foreign body, such as a fish bone, out before it. For the removal of larger foreign bodies œsophageal forceps of different patterns are employed some opening laterally, and others antero-posteriorly, (Fig. 4).

Stomach Pumps, &c.—Numerous arrangements have been

Fig. 2.

Fig. 3.

Fig. 4.

devised to wash out the stomach, such as that shown in Fig. 5, but perhaps the simplest and safest is an ordinary soft rubber tube fitted to a metal or vulcanite funnel and acting on the syphon principle. Such an apparatus may also be used for feeding lunatics and others who refuse to swallow food. The tube for this purpose may be passed either through the mouth or nose.

Rectal Instruments.—(1) Specula are used to separate the walls of the rectum so as to admit rays of light into the cavity to enable the parts to be examined, either for purposes of diagnosis or treatment. Direct sunlight or light reflected from some artificial source may be employed. Many forms of instrument have been introduced—some tubular, for example, Fergusson's (Fig. 5A); others with two separate blades, such as Maw's (Fig. 6), and still others with three and four blades like Allingham's. (2) Rectal bougies are employed to detect the situation and degree of strictures of the rectum, and for gradual dilatation of these when such is possible. They are graduated to a scale, are usually made of soft gum-elastic, and the points may be cylindrical, conical or bulbous. (3) Rectal dilators are used to distend the tube more forcibly, the blades being separated by various mechanical arrangements. Fig. 7 illustrates one of these introduced by Todd. (4) Rectal bistouries, for the purpose

Fig. 5.

of operating on such conditions as fistula in ano, have been introduced, but are for the most part unnecessary.

In the treatment of hæmorrhoids several special instruments are used. (a) Hæmorrhoid forceps (Fig. 8), which are used to seize and pull down the hæmorrhoids. (b)

Fig. 5a.

Fig. 6.

Fig. 8.

Fig. 9.

Hæmorrhoid clamps.—Of these there is infinite variety. Some are used in conjunction with the cautery. *e.g.*, H. Smith's and Sydney Jones's (Fig. 9), a plate of ivory being placed between the metal and the skin to prevent the latter being burned. Others crush the piles sufficiently to prevent hæmorrhage when they are cut off by scissors, *e.g.*, Allingham's (Fig. 10). (c) Cauteries.—Any form of cautery will do for treating piles, although several have been specially contrived for this purpose. (*d*) A Hæmorrhoid needle is a long, sharp, curved needle fixed in a handle, and having the

FIG 7. FIG. 10. FIG. 11.

eye a short distance from the point. It is used to carry a double ligature through the pile, which is then tied in two halves (Fig. 11). Others are made after the pattern of cleft palate needles.

The illustrations are used by kind permission of Messrs. Arnold and Sons, and Messrs. Maw, Son, and Thompson.

### Appointments.

LONDON HOSPITAL.—Miss Martha Reatley, who trained at the London, has been appointed night Sister at that institution.

HONG KONG.—Miss Caroline Walker, who trained at the London Hospital, and has lately worked as a Sister at Gibraltar, sails on the 25th for Hong Kong, to take up the duties of Sister in the Government Hospital there.

MANCHESTER AND SALFORD DISTRICT NURSING ASSOCIATION.—Miss Cox, Night Superintendent, Monsall Hospital, has been appointed Superintendent of the Hulme Branch of this Association. Miss Cox, who was trained at the Adelaide Hospital, Dublin, has had several years' experience in district nursing in the north of London. We congratulate the Association on having secured her services, and wish Miss Cox every success in her new work.

### Presentation.

THE nurses of University College Hospital on February 11th presented the Secretary, Mr. Newton H. Nixon with a handsomely fitted luncheon basket, on the occasion of his birthday. We understand that the idea originated with the nurses whilst returning from their last summer trip to Richmond annually organized by Mr. Nixon.

### Wants and Workers.

*Linen or Gold?*—Will anyone help a cottage hospital, either with new bed and house linen, or with subscriptions to aid our repairs?—*Matron, Cottage Hospital, Warminster, Wilts.*

*Help Received.*—Miss Harriet Middleton records with thanks that she has received an offer of £10 a year in answer to her appeal for funds to get a nurse out of the workhouse. Further help needed.

### ROSE-COLOURED CURTAINS.

A GREAT humourist some years ago held up to derision the vanity of an old lady who was struck down with paralysis, and on her partial recovery of speech used the first words she could utter in giving the order "Rose-coloured curtains for the doctors." Poor soul! she had been a great beauty in her day, and the ruling passion was strong in death, she wished to look her best to the last.

Vanity at any time is a foolish and a hateful weakness which leads people astray in many ways, while in illness it is useless to "put on parts" or lay ourselves out for effect. We can, however, gain a bright idea from the little story above while we avoid its errors. For instance, we can make the best of our sad case by hanging up moral and mental "rose-coloured curtains" around our beds for the benefit not only of the doctors, but of the nurses and ourselves. Curtains are often hung up by our own hands, though unfortunately they are apt to be of a wrong shade. If we choose yellow we get a jaundiced view of life, as everything looks glaring and uncomfortable; we are jealous, perhaps, of our friends' good health and success in their undertakings, and imagine all sorts of foolish slights which are never intended. If we use green curtains, they shut out too much light and life and we become morbid and melancholy. The worst sort of curtains are dark thick ones of any colour which keep out all light; some of us fasten these closely and securely around us, and struggle and fight against any attempt to remove them. But the sooner we take them down the better, for even the Son of Righteousness cannot pierce the gloom when we have determinately set our faces like a flint to make the worst of our lot and take everything in ill-part.

But if by nature or still better by grace we hang up pretty rose-coloured curtains about us, what a transformation takes place, every view is warm and bright and comforting in the atmosphere of love and thankfulness. Our hearts can respond to every working of the Holy Spirit which prompts gratitude to our Heavenly Father for the kindness and help we receive from His children, our fellow creatures. Who can be unhappy or repine, when the rich soft folds of a Saviour's love close around us? Our sufferings will appear light with the love in our hearts which beareth all things, even the unkindness of those most dear to us which thinketh no evil of the most trying people, which endureth all things for the love of Him who loved us and gave Himself for us. This love will spread out from ourselves and wrap up all who come near us, and in their turn our attendants and friends will be cheered and refreshed by the ready smile we give, the bright view we take of things, the patience with which our troubles are borne.

### District Nurses Under the Poor-Law.

A new and important scheme for district nursing has just been promulgated by the Local Government Board who have issued a circular and order to all the Boards of Guardians throughout the Kingdom. The Local Government Board having had their attention drawn to the frequent absence of suitable persons to attend on sick poor receiving out-door relief in this order empower Guardians to appoint persons to act as district nurses, and that these persons should have a recognised position the Board issues certain articles on the subject.

Article I. of the Order authorises the appointment by the Guardians of the Poor of any union or separate parish, with the Board's approval, of one or more officers, who are to be termed district nurses, and Article II. makes applicable to the mode of appointment, remuneration, and tenure of office of a district nurse, the provisions as to those matters which are in force in regard to the appointment of a nurse at a workhouse. The Board consider that it is of great importance that the persons who may be appointed by Guardians to the office of district nurse should have had thorough practical training in the nursing of the sick; and Article III., therefore, directs that no person shall be appointed to the office who has not undergone, for one year at the least, a course of instruction in the medical and surgical wards of a hospital or infirmary being a training school for nurses and maintaining a resident physician or house surgeon. A longer period of training than one year would seem desirable, although the Board have not deemed it expedient to insist upon it as an indispensable condition. The Board have by Article IV. of the Order, directed that it shall be the duty of a district nurse to attend duly and punctually, as a nurse upon any poor person or persons in receipt of medical relief, when directed by the Guardians, or upon receipt o a printed order from a relieving officer in any case in which that officer may be authorised by regulations to be prescribed by the Guardians, to give such order. A district nurse is also to obey any directions of the District Medical Officer in attendance upon any poor person, in regard to the nursing and treatment of such person, and to keep a record, in a form to be determined by the Guardians, of the cases which she attends. Apart from these prescribed duties, and subject to the prohibition contained in Article V. that no district nurse shall undertake the duties of a midwife, the Board have left the duties of the district nurse to be settled by regulations which, under Article VI., the Guardians are required to make. The last Article (VII.) merely defines the various expressions used.

Undoubtedly this is a very practical and well-thought out scheme, and is the most important piece of nursing news issued for many a long day. There are certain difficulties and dangers before the coming Poor Law nurses, who will have to be very careful not to interfere with existing societies, or to let their work overlap that done by the Queen's nurses. It will almost become a question now whether the poor should not pay a small sum, were it only a penny a-week, for the services of ordinary district nurses. In small towns where no district nurses yet exist the Order might be of the greatest use, only there is the danger of apathy and ignorance on the subject, especially where the Guardians are all men. One case of this sort has already come to our notice. The Brentford Board, whose ignorance on nursing matters is only equalled by their indifference, according to a local paper thus received the above Order: "Some laughter was created by a long letter from the Local Government Board, the purport of which was to empower the Board to provide nurses, at £1 a week, to relieve the poor under exceptional circumstances. The Board directed the clerk to reply acknowledging the receipt of the letter." However, the Local Government Board has means of retaliation on those who only laugh at its orders, and the public are not likely to let an important plan like this "fast unused." Our readers will doubtless have something to say about the scheme, especially those who are lady Guardians, or who are engaged in district work.

### Everybody's Opinion.

*[Correspondence on all subjects is invited, but we cannot in any way be responsible for the opinions expressed by our correspondents. No communications can be entertained if the name and address of the correspondent is not given, or unless one side of the paper only be written on.]*

### NORTH LONDON NURSING ASSOCIATION.

Miss de Luttichav writes: Not long ago an advertisement appeared in your columns, in which a home at Holloway was mentioned as a "branch" home of the Metropolitan and National Nursing Association. Since this statement is misleading, may I be allowed to correct it by explaining that the Home at 413, Holloway Road, was opened as the first branch of the Metropolitan and National Association in January, 1877, but that since February, 1881, the work has been carried on by a local Committee, under the name of the North London Nursing Association, being in no sense a "branch" of any other institution. This Association for district nursing is doing the largest work of the kind in London. The nurses it employs are gentlewomen, well trained and educated in their work, and great care is taken to maintain the nursing at a high point of efficiency, as well as to give to those who desire it a thorough training in district nursing. At this moment more workers are urgently wanted for the continually increasing work of the Association. Salaries begin at £35 and rise to £50 per annum. It must be added that the North London Nursing Association is not affiliated with the Queen Victoria Jubilee Institute.

### A NURSING RESERVE.

"Red Cape" writes: When a country is threatened with invasion by a hostile people, there is a general call to arms of forces on the active list, and if the emergency be great, those on the reserve as well. Now for the last three years we have been suffering much at the hands of an invading foe (influenza), and have generally found our forces inadequate to repel the enemy. Might not a scheme be arranged for a reserve of nurses on the same lines as those followed by the army and naval authorities for strengthening the hands of their soldiers and sailors? The nursing reserve might consist of retired but fully-trained nurses receiving a small yearly sum, while following their own pursuits, but liable to be called upon by the hospital or institution to which they are attached for active duty with full pay. This is quite a rough sketch of an original idea, but I should like to hear if it is thought practicable.

### ONE FUNERAL MAKES MANY.

"A Scotchwoman" writes: A funeral ceremony is a recognised source of danger, and it is easy to account for the fact. The vitality of a mourner is necessarily low, the associations of the churchyard are of the most depressing character, while a fruitful source of active mischief may be the grave itself, the fresh turning up of earth which is presumably not free from noxious gases. The fact that these natural or incidental risks are rendered more dangerous to the mourners by exposure to cold during the service at the grave proves the need for some reform in burial customs. It were surely quite seemly that the religious exercises be conducted under cover in church, and service at the grave limited to a short prayer, as is done in Scotland, where, however, in many cases even this ceremony is omitted. The solemn silence is not without its impressiveness; surely such an occasion, for even the hardened and worldly must "give pause" in the immediate presence of death, requires nothing in the shape of ritual to heighten its solemnity. The objection to change is chiefly a sentimental one, and none the less difficult to overcome on that account. The argument of "use and wont" is ever powerful.

### Memoranda for Matrons.

THE following are some scattered hints on the duties of Matrons, and particularly on housekeeping points; but a whole treatise might be written on the subject and would be a welcome volume to many. There are two books already existing which are of use, namely, "Hospital Sisters," by Miss Lückes, and "Handbook for Hospital Sisters," by Florence Lees, but neither treat of the actual housekeeping. True, Miss Lees has some excellent chapters on the care of linen, &c., but the only article on direct domestic management is by Florence Nightingale in "Quain's Dictionary."

The Matron in every well regulated institution has full control of all the nurses and female servants, subject only to the weekly board; her chief duty then is to appoint competent women under her, and to keep a careful watch over them and their work; finding fault whenever necessary, but also giving praise at times in order to cheer and encourage the good workers. But you cannot teach what you do not know—you cannot direct and control on points of which you are ignorant. Therefore, a Matron must be acquainted with the best means of procuring food by contract, and cooking and serving food by quantities. She must, when going round the kitchens, see that the coppers are clean, the larders are sweet, and all the sinks and out-houses free from smell. She must know the best means of washing greasy sheets, the rules for washing infected linen, the difficulties of dealing with old linen. She must give all her subordinates written orders and written time-tables, and see that servants as well as nurses have their regular meal times and hours off duty. She must in return get written daily reports from every department, and before going off duty every night must fill in her own diary and petty cash book and file the other reports. Everything must be brought before the weekly Board at its meeting, and the Matron must be ready to seek advice, and submit plans for improvement, or any complaints to the Board. For a knowledge of small domestic details no training is so good as that got in a middle-class home where the daughters help in the house-work. Of course, in an institution where food is supplied by contract, and cooking is done by gas or steam, things are very different to what they are in a private house; but once having learnt to know good meat by its appearance, and to judge by taste where the cooking is defective, the knowledge can be applied to things great as well as to things small. Very low contracts are not to be recommended, as it is poor economy to procure bad food at any price; it is useful to compare the prices paid by other hospitals, which can generally be found in the published reports. Comparing the cost per bed or per head is not of much use, as it is calculated on different lines in different places. The market prices as quoted in the daily papers should be studied, and from the nurses and officers' tables hints can be gleaned from the little provision paragraph given every Saturday in the *Daily News*; there are officers and nurses' tables which go from year's end to year's end without celery because this cheap and wholesome vegetable is not on the contract list, though it can be bought at times for a penny a-head from any greengrocer. Again, marmalade, a cheap and wholesome addition to the breakfast table, costing only 9½d. for three pounds, is seldom seen in institutions in the South; and Scotch broth, a nutritive and economical dish, is known only in the country from which it gets its name.

Method and management are the two chief virtues needed by a Matron, and she must arrange her time carefully so as to give due attention to every detail of the big household over which she reigns. She must receive each Sister at a certain hour, also the housekeeper or cook, and she must visit every part of her domain during each twenty-four hours. She must see that there is no waste or wilful damage, and keep inventories of all the goods under her care. There should be a quarterly stocktaking in every department.

The nurse who has just gained her first matronship should not hesitate to seek advise from Matrons of long standing; asking for their rules and for a list of the books they keep, and how they portion out their daily round. But the duties of Matrons vary in every hospital, and each has to form her own plan in accordance with the wishes of the Committee under which she works. It is a great point to have a clear definition of all duties, so that each officer goes his or her way in a clearly-marked limit, and the less over-lapping there is, the less quarrelling there will be. A little patience and courtesy helps to oil the wheels also, and new brooms must not be in too great a hurry to sweep clean.

### The Mortality of German Catholic Nurses.

IN Professor Tyndall's very interesting series of papers, called "New Fragments," just published, he gives some striking and important statistics regarding the mortality of nurses, specially from tubercular diseases. The figures are abstracted from Dr. Cornet's researches on the communicability of phthisis, which were published in the fifth and sixth volumes of the *Zeitschrift für Hygiene*. Cornet's enquiry extended over a quarter of a century. The returns furnished by 38 Prussian hospitals, served by Catholic sisters and brethren, and embracing a yearly average of 4,020 attendants, showed the number of deaths during the period mentioned to be 2,099; of these, 1,320 were caused by tuberculosis. It is further stated as to dangers incurred by nurses that a healthy girl of 17 devoting herself to hospital nursing dies on the average 21½ years sooner than a girl of the same age moving among the general population. A hospital nurse of the age of 25 has the same expectation of life as a person of the age of 58 in the general community. The age of 33 in the hospital is of the same value as the age of 62 in common life. The differences between life-value in the hospital and life-value in the State increases from the age of 17 to the age of 24; nurses of this latter age dying 22 years sooner than girls of the same age in the outside population. The difference afterwards becomes less. In the fifties it amounts to only six or seven years, while later on it vanishes altogether. The reason of this is, that the older nurses are gradually withdrawn from the heavier duties of their position and the attendant danger of infection. It is the younger nurses, engaged in the work of sweeping and dusting, whose occupation charges the air they breathe with the virulent bacilli of tubercle, who fall victims to phthisis in the blossom of their years. In these hospitals also deaths from typhus and other infectious disorders exhibit a frequency far beyond the normal. The statistics of the mortality of young Catholic nurses Cornet regards as a monumental record of their lofty self-denial, their noble, beneficent, and modest fidelity to what they consider as the religious duty of their lives. The Protestant nurses, owing to the greater freedom of their lives, to changing their occupation, or to entering the married state, suffered less; and in considering the mortality of nurses Dr. Cornet confined his attention solely to the nurses of the Catholic orders.

These nurses are constantly and permanently engaged at the work of nursing, and it would be interesting to compare the melancholy statistics furnished by Dr. Cornet and quoted by Professor Tyndall with those furnished by such an association as the Royal National Pension Fund, the nurses of which probably carry on their vocation under regulations and with precautions more strictly sanitary and scientific than the nurses of the Catholic Sisterhoods of Germany.

### Saint and Sinner.

At the best of times it was not an inviting neighbourhood, but in the dull grey mist of that November afternoon it surely typified "The Abode of Desolation," and the gaunt hunger-stricken creatures who thronged its narrow courts, the denizens thereof. Few would have risked contact with them voluntarily, but the slim figure in the long grey cloak passed through them unmolested. For the most desperate had a half grateful, half superstitious reverence for "Sister," who seemed to them like some one from another sphere. She was a little hard on them perhaps, but then as one said once: "Sister's such a saint herself that she doesn't know what it means to the likes of us to be tempted."

Their voices were hushed to-day, for close by, in one of the gloomiest of those gloomy dens, Jim Pointzer was dying. A rough dare-devil sort of fellow, he had cared neither for God nor man, and had only been kept from utter lawlessness by the love of one poor creature, whose faithfulness was all she had to redeem her from the lowest depths. It was to him that Sister Alice was going, but once there, she stood unheeded in the doorway. For Jim's fast dimming eyes had no sight save for the kneeling figure beside him, and it would have taken more than the Sister's gentle knock to reach those ears just then.

"Nell, old woman," he was saying, the hoarse voice sounding strangely pathetic in its weakness, "Nell, I've been a brute to you often, but you—you've struck to me through thick and thin—and I'd been a worse fellow if it hadn't a been for *you!*"

A flood of light transfigured the poor wan face into absolute beauty as she heard him. "I've only *loved* you, dear," she whispered brokenly, "that's all, Jim."

"*All?*" he answered. "Ah, well, if *God* forgives—as a *woman* does—then there's a chance for me yet."

"As a *woman* forgives." How the words haunted Sister Alice as she passed out into the mist again. *Her* "forgiveness" had been a proud withdrawal from contact with what she termed "unworthy," and sometimes as the loneliness of her life pressed heavily upon her, she wondered drearily if after all she had been right. "Perhaps, if I had been more patient," she thought bitterly.

A carriage drove up immediately behind her, and some one spoke her name.

"Sister Alice? How fortunate! Can you spare an hour or two to a poor fellow in Hallow Street who is dying, with no one beside him but an old landlady? Yes? Then jump in—I'll drive you round. Its too late to do anything for him now, but just stay with him till it's all over. It's an awfully sad story" the doctor went on, half to himself, as they drove quickly through the streets. "I knew him well by reputation ten years back. A splendid fellow he was, too, but just the kind that needed a good woman to keep him straight. And his wife left him within a year of their marriage, I believe—some bye-gone folly of his came to her ears, I fancy, and she was too much of a saint to look over it, and he threw everything up then, and went to the devil. A pity—a sad pity," he added, the kindly face growing stern and set as he said "Ah, Sister, your sex has a deal to answer for!"

A moment later they reached the house. "Will you go straight up?" he asked. "Third floor, first door to your left. I'll be back as soon as possible." And he drove rapidly away, with a passing wonder that she should have trembled so as he helped her out of the carriage. "Nervous, poor thing," he thought. "Overdone, I suppose."

It was with an odd thrill of foreboding that Sister Alice found her way upstairs and into the room he had indicated. She almost knew what she would see there—the strangely familiar face that even fever could not rob of its rugged beauty, and the stern deep-set eyes that had looked into her own such volumes of tenderness or anger in days gone by.

"Alice, is it you?" he asked slowly, the parched lips twitching into the grim semblance of a smile. "Ah well, it is fitting that my wife should be with me now!"

Silence—and then the pent-up agony of remorse that had been with her all those years found vent in words, and the love and tenderness that might have saved him were his at last.

But, as so often happens, it had come too late. "You," he cried—and the scorn in the weak voice went through her like a knife—"you, with your idealisms and hero worship, care for a man who has sunk to depths like mine? . . . . Ah!" he murmured, touching her bent head softly, "it was there the mistake came, Alice. I did not want to be thought a hero; I was but a mortal man after all, with stronger passions than most. I fancy so? You are crying, dear. Ah, but its over now . . . . . and you were quite right, you know!"

And that was all. For the brief gleam of consciousness vanished, and in a little while the storm-tossed soul has ceased its wanderings, and was at rest at last.

And Sister Alice was alone again, with only vain remorse for company, and a deep envy of that other woman, whose love, unlike her own, had stood life's test.

"Saint and Sinner," some one muttered a few days later, as he saw them standing together beside an open grave, and the winter sunlight shining upon them both, and bringing into strong contrast the pale pure face of the one and the passion-worn countenance of the other. "Saint and Sinner" he called them; but which, in God's sight, was the truer woman?

### Where to Go.

St. John's Ambulance Association.—A ladies "nursing" course will commence on February 25th at 2.30 p.m. at 25, Inverness Terrace, Kensington Gardens. Fee 10s. Tickets can be had from the Hon. Secretary, 11, Ludgate Hill, E.C. All young women waiting to enter hospitals should go through the St. John's course.

### Notes and Queries.

### Query.

*Home Wanted.*—Free, for an incurable woman. She is solely dependent on a sister, a servant.—*Agnes.*

### Answers.

*Dolly.*—We never prescribe. Consult a medical man.

*H. Gibraltar.*—All the military hospitals of India, for appointments in which application should be made to the Under Secretary of State, India Office, Whitehall. The Hyderabad Hospital is under Miss Lawrie, late Sister at the London; write to her direct. Miss Henan is Matron of the Madras Hospital for Women and Children. We must really refer you to the "Hospital Annual" for further particulars.

*Enquirer.*—Write to the Matrons or the secretaries of the various hospitals mentioned in the "Hospital Annual," or if you would like private nursing in Sydney write to Lady Samuel, Courtfield Gardens, Earl's Court, S.W.

## Figures and Tables

**Fig. 2. f1:**
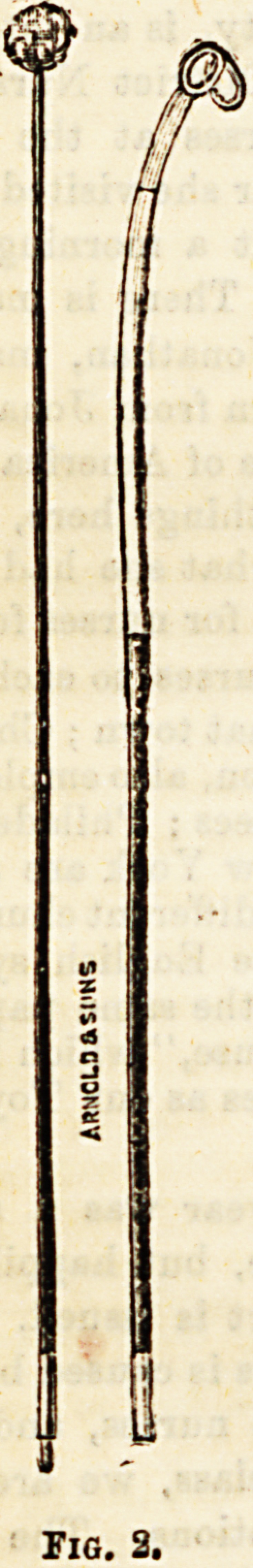


**Fig. 3. f2:**
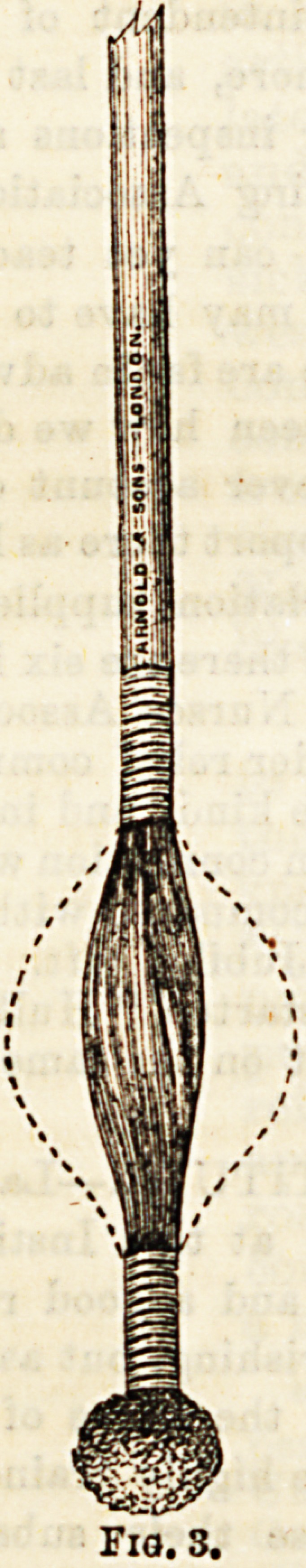


**Fig. 4. f3:**
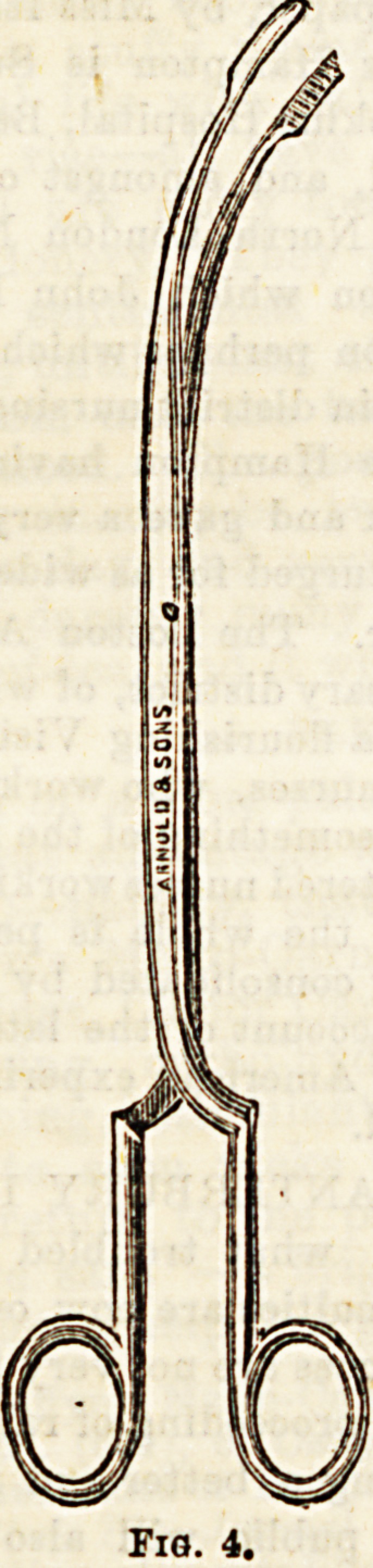


**Fig. 5. f4:**
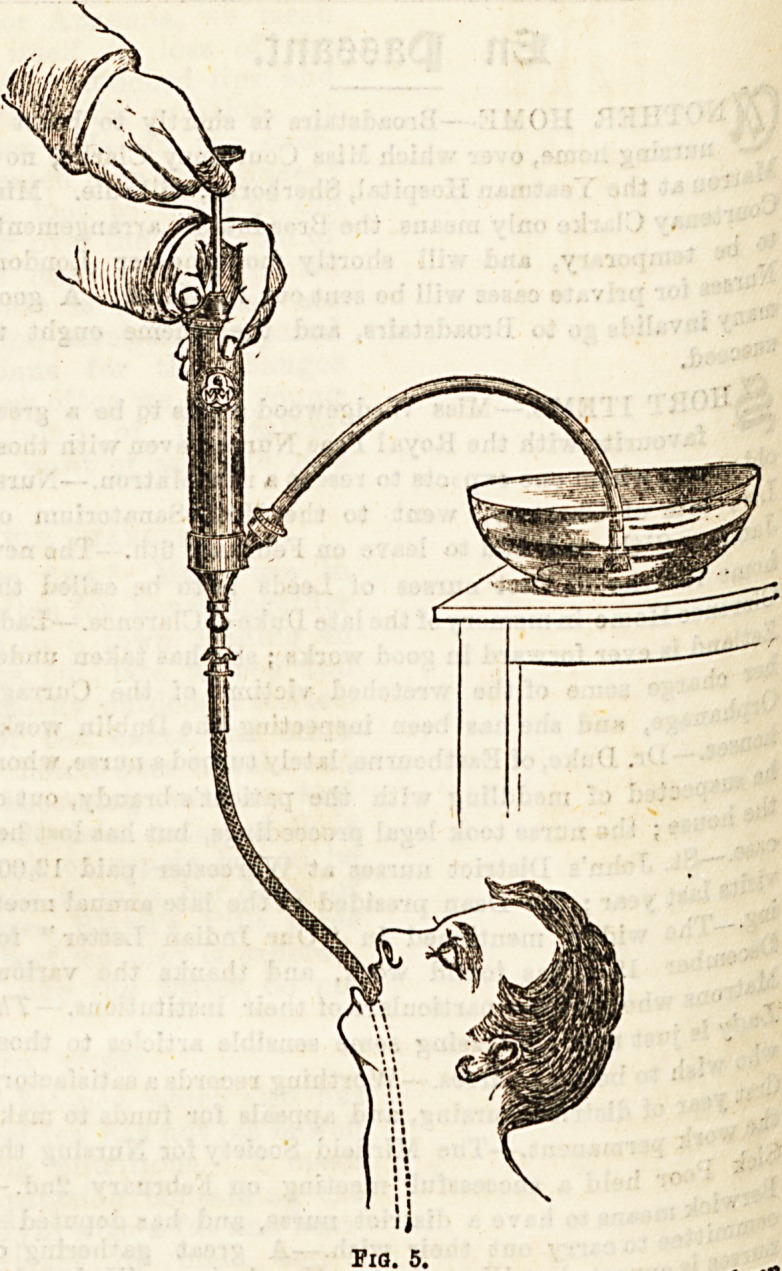


**Fig. 5a. f5:**
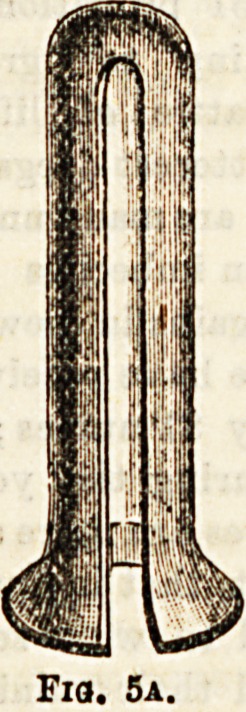


**Fig. 6. f6:**
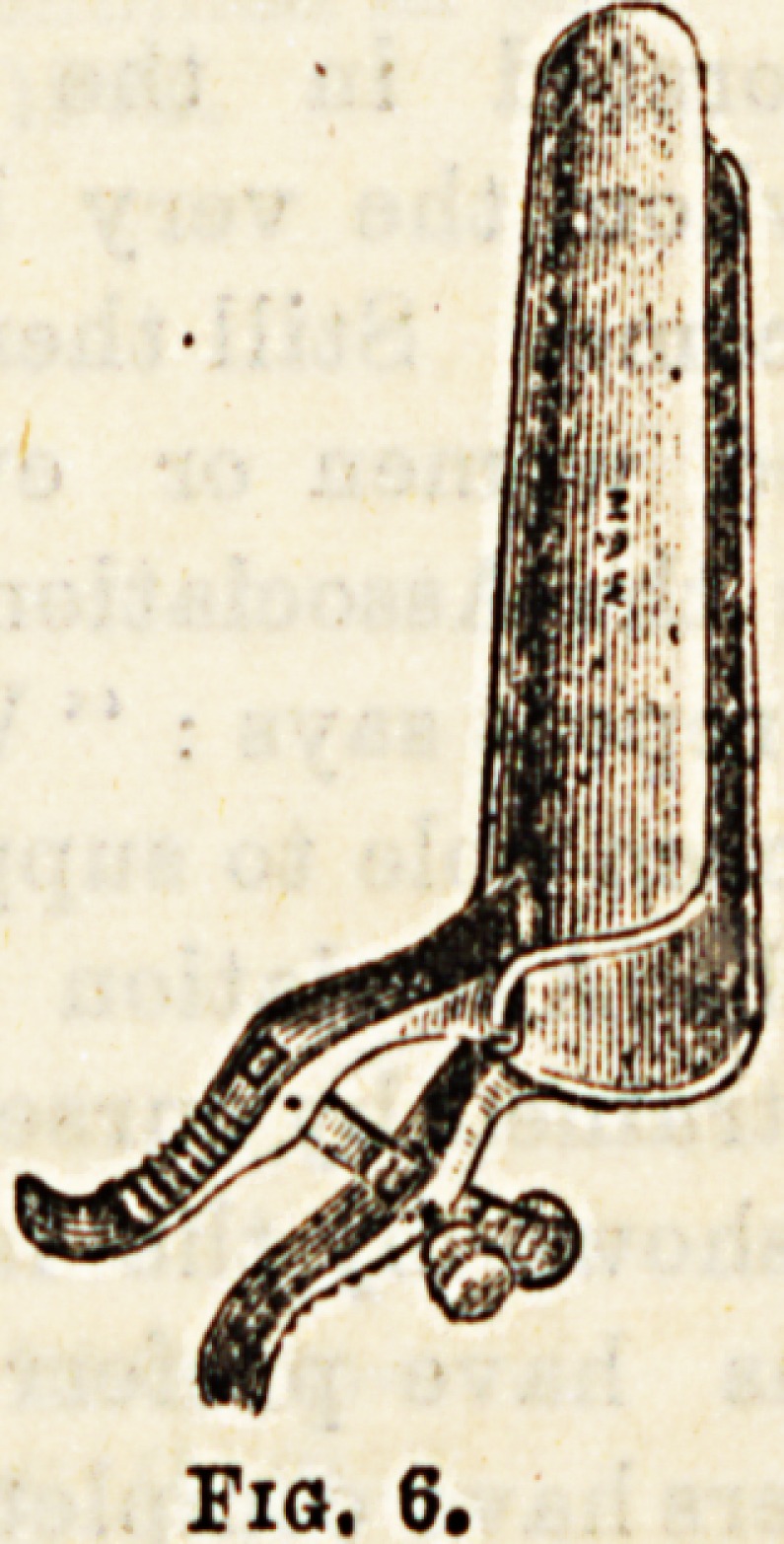


**Fig. 8. f7:**
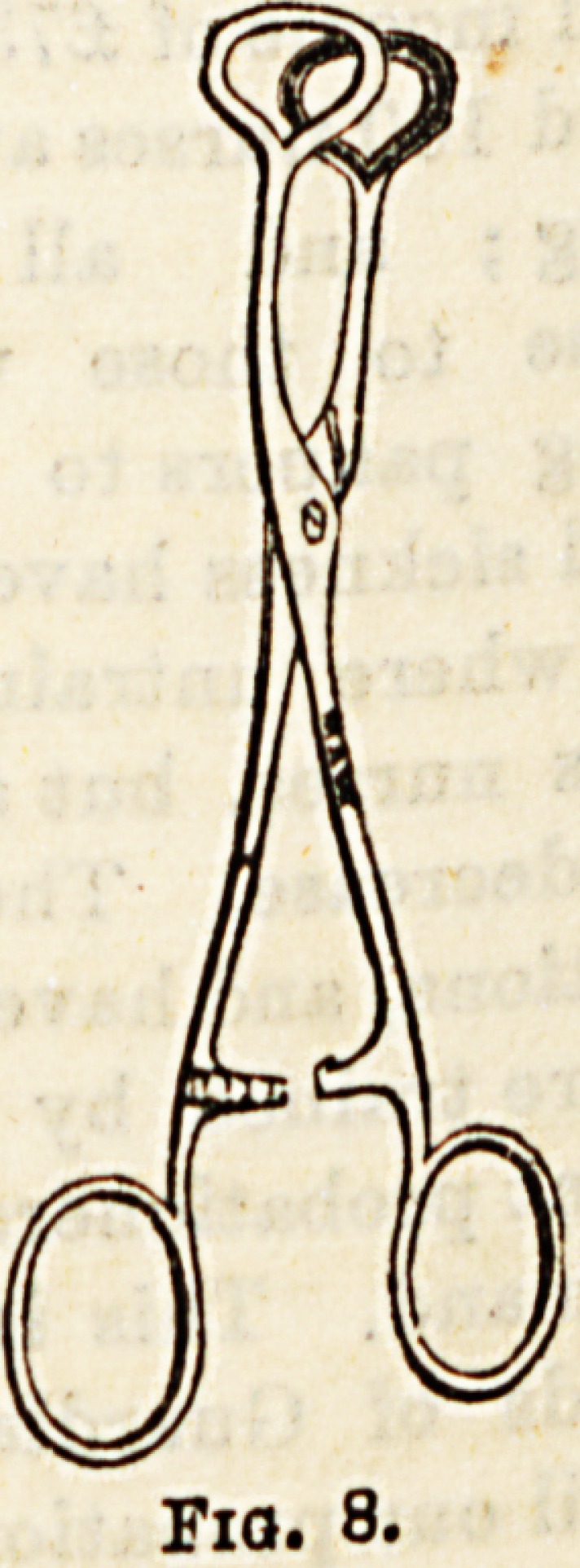


**Fig. 9. f8:**
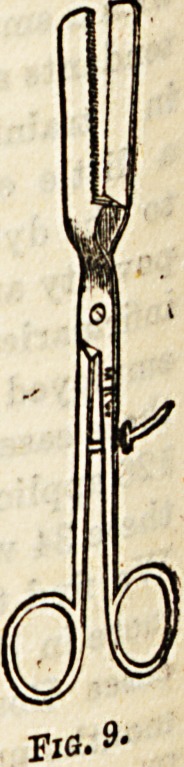


**Fig 7. f9:**
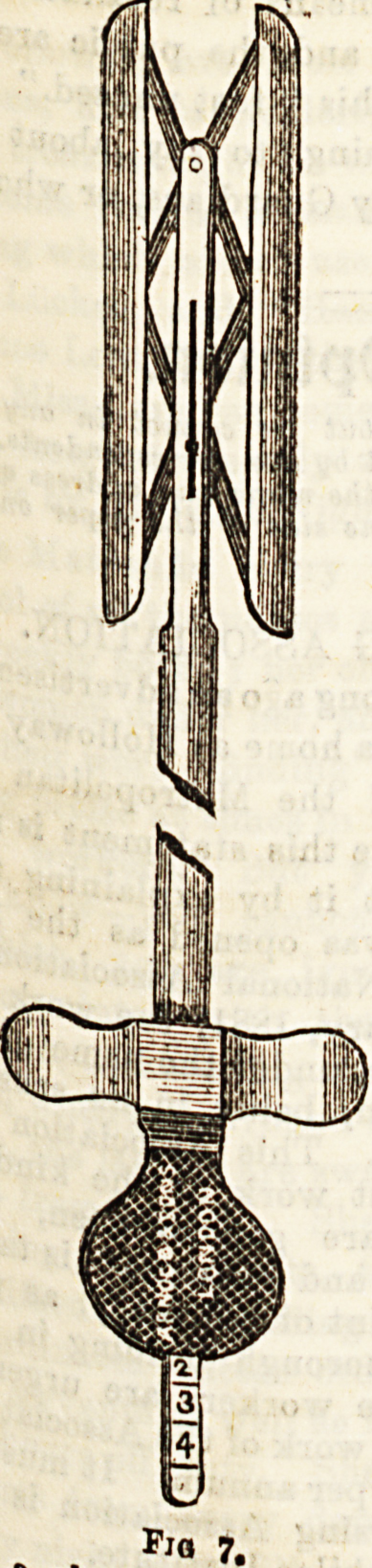


**Fig. 10. f10:**
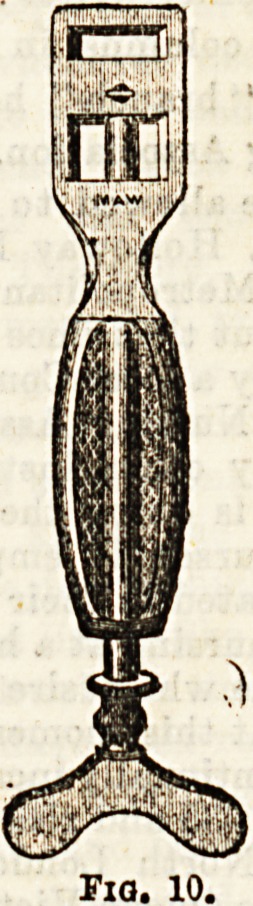


**Fig. 11. f11:**
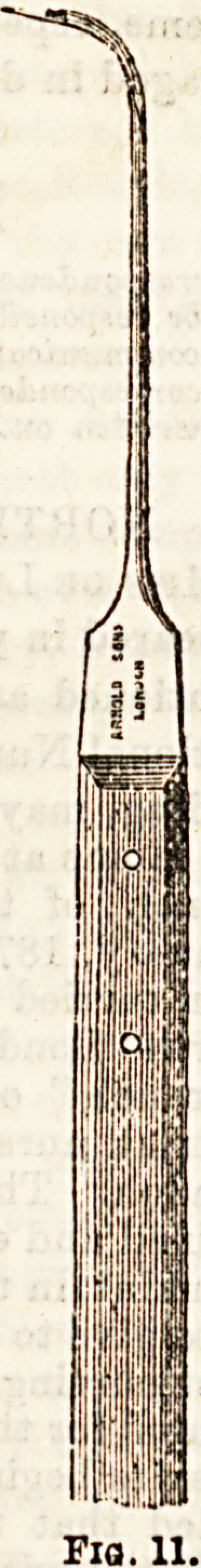


**Figure f12:**